# Complete Genome Sequence of a Jumbo Bacteriophage, vB_pir03, against Vibrio harveyi

**DOI:** 10.1128/MRA.00910-20

**Published:** 2020-10-29

**Authors:** Gerald N. Misol, Constantinta Kokkari, Pantelis Katharios

**Affiliations:** a Institute of Marine Biology, Biotechnology, and Aquaculture, Hellenic Center for Marine Research, Heraklion, Crete, Greece; b Department of Biology, University of Crete, Heraklion, Crete, Greece; Queens College

## Abstract

Vibrio harveyi is a persistent pathogen responsible for disease outbreaks in aquaculture. We have sequenced the genome of a jumbo *Vibrio* phage, vB_pir03, isolated in Greece. Here, we present the complete genome of vB_pir03, which consists of 286,284 bp and 336 open reading frames.

## ANNOUNCEMENT

Vibrio harveyi is a persistent bacterial pathogen in aquaculture that causes vibriosis in marine finfish, crustaceans, and mollusks (1 – 3). The emergence of antimicrobial resistance in aquaculture has prompted the search for alternatives; one of the most promising is phage therapy, which is the use of bacterial viruses as therapeutics (4 – 9). Here, we report the complete genome sequence of vB_pir03, which was isolated in Piraeus, Greece (37°56′49.7″N, 23°38′29.5″E), against the Vibrio harveyi type strain DSM19623.

In brief, the phage was amplified using concentrated LB and host bacteria and was plated on LB-top agar at 25°C. The phage was then purified through six successive single-plaque isolations. The phage titer was amplified by liquid propagation until it reached approximately 10^9^ PFU/ml for DNA extraction. The DNA was extracted according to the phenol-chloroform method described previously (10). Both library preparation for the BGISeq-500 sequencing system (11) and whole-genome sequencing using the BGISeq-500 sequencing system (Beijing Genomics Institute [BGI], Shenzhen, China) (12) were performed at the BGI in Hong Kong. The quality of the reads was assessed using FastQC v0.11.9 (13). The reads were *de novo* assembled with Unicycler v0.4.8 (14) using the Pathosystems Resource Integration Center (PATRIC) v3.6.5 Web server (15). The reads were mapped back to the assembled genome using QUAST v4.6.3 (16) and BBMap v38.88 (17). PhageTerm was used to predict phage termini (18) through the Galaxy server (19). Predicted open reading frames (ORFs) were called using RASTtk (20), Genemark.hmm v2.0 (21), and Glimmer (22). The genome was analyzed for tRNAs using ARAGORN (23) and tRNAscan-SE (24). Predicted ORFs of vB_pir03 were searched against the NCBI nonredundant database using BLAST (25). Default parameters were used for all software unless otherwise specified.

Sequencing of vB_pir03 produced 41,500,540 clean reads with an average read length of 150 bp and a GC content of 43.6%. The per-base call scores produced a median score of 36, while the proportions of the four bases remained relatively constant throughout the read length, with the percentage of A being equal to that of T and the percentage of G being equal to that of C. The GC contents of all reads formed a normal distribution with no deviation of the peak of the curve from the theoretical peak. Per-base N content results showed that no N substitutions were made. Unicycler assembled the genome of vB_pir03 into a single contig with a minimum genome coverage of 5×. The total genome length of vB_pir03 was 286,284 bp, which indicated that it is a jumbo phage. A total of 99.91% of the raw reads were mapped back to the assembled genome, resulting in an average coverage depth of 21,669×. In addition, the vB_pir03 genome was predicted with PhageTerm to be circularly permuted. A total of 336 ORFs were predicted in the genome of vB_pir03, with 68 ORFs showing the greatest similarity to another jumbo *Vibrio* phage, vB_BONAISHI (GenBank accession number MH595538), which infects Vibrio coralliilyticus (26). No tRNA genes were found in the genome of vB_pir03.

### Data availability.

The genome sequence of phage vB_pir03 is available in GenBank under accession number MT811961. The associated BioProject, SRA, and BioSample accession numbers are PRJNA665717, SRR12712979, and SAMN16261552, respectively.
